# Interpersonal sensemaking and cooperation in investigative interviews: The role of motivational matching

**DOI:** 10.1177/17470218251348932

**Published:** 2025-06-02

**Authors:** Mattias Sjöberg, Paul J Taylor, Stacey Conchie

**Affiliations:** 1 Durham University Business School, Durham, UK; 2 Lancaster University, Lancaster, UK

**Keywords:** Investigative interview, interpersonal sensemaking, motivational frame matching, cooperation, cylinder model

## Abstract

The cylinder model of interpersonal sensemaking predicts that cooperation emerges in interactions where speakers are matched on motivational frames and cooperative rather than competitive in orientation. The purpose of the current study was to provide the first evidence of a causal link between motivational frame matching and cooperation and trust in an investigative interviewing context. Over two pre-registered experiments (*N* = 776), participants took the role of a suspect during an interaction with an interviewer. During the interaction, the interviewer and suspect either matched motivational frames (in an instrumental, relational or identity motivational frame) or not, in either a cooperative or competitive way. It was found that within a cooperative orientation interaction, motivational frame matching led to significantly higher willingness to cooperate and greater feelings of being understood among the participants. In contrast, within a competitive orientation interaction, motivational frame matching led to significantly less willingness to cooperate and identify with the interviewer.

As countries move away from accusatorial to information gathering approaches of investigative interviewing (Meissner et al., 2017), several constructs have been highlighted as leading to greater cooperation and better information elicitation. These include rapport, perspective taking, empathy and active listening (Russano et al., 2019). While all important, there has been less focus on how interviewers supposedly make sense of suspects’ communication and how they may respond appropriately. This phenomenon, termed interpersonal sensemaking (Taylor, 2002; Taylor & Donald, 2004), is an important skill for anyone who engages with people, such as interviewers, because it provides the foundation for inferences about the other’s intent and decisions about how to respond. Across two experiments, involving both text-based and video-based stimuli, the current study examines sensemaking in investigative interviews. It builds upon previous correlational work on sensemaking to test experimentally whether effective sensemaking of speakers’ motivations leads to more positive interaction outcomes.

## Sensemaking in investigative interviews

In the context of police interactions, interpersonal sensemaking refers to the ability of the interviewer to make sense of the motivations and goals that underpin a suspect’s behaviour (Arnold, 2021). For instance, while an investigative interviewer likely wants a suspect to provide as much information as possible, the suspect may be more interested in telling their story of what led up to the incident and the reasons for why it happened. A skilful sensemaker would recognise the discrepancy in motivations between the speakers and try to better align them (i.e. match motivations; Wells & Brandon, 2019). The acknowledgement of the suspect’s motivation could be sending a signal of consideration and being respected (Ury, 1991), helping to facilitate a process of cooperation and mutual understanding. Successful interpersonal sensemaking may also constitute the first building block of a shared reality between interaction partners (Rossignac-Milon et al., 2021), where they start to see and experience things in similar ways. In the current research, successful interpersonal sensemaking is conceptualised as adopting similar goals and motivations within an interaction (i.e. motivational frame matching).

The majority of research on interpersonal sensemaking has focused on crisis negotiations, where Taylor (2002) identified instrumental, relational and identity motivations as three common frames for engaging in an interaction.

Suspects in an instrumental frame would mainly be focused on the concrete problems at hand and motivated to solve them. Statements that are framed around instrumental goals or motivations often relate to the transmission of facts and information or exchanges of wants and needs. For example, a suspect telling a police interviewer when they last saw the victim in person would be communicating in an instrumental motivational frame. Several theories, such as the social exchange theory (Cropanzano & Mitchell, 2005) and dual concern model (Blake & Mouton, 1970), have highlighted that many interpersonal interactions tend to be influenced by their transactional nature. In the investigative interview space, the cognitive interview is an example of an instrumental interview technique where the suspect is encouraged to remember and report everything they can remember, without ignoring any details (Fisher et al., 1989). From an interviewer’s perspective, having a suspect communicate in an instrumental frame might be helpful for obtaining insights that can later be used as evidence in court.

In contrast to the instrumental frame, relational motivations are about either establishing or breaking down the relationship they have with another person (e.g. how interaction partners negotiate and manage their relationships). The importance of relational motivations such as trust and power during high-intensity interactions, including tough negotiations, was recognised early in work by Greenhalgh (1987). Others have looked at the different stages that a relationship goes through from first meeting another person to the eventual dissolution of the relationship (Knapp & Vangelisti, 2009). In a similar vein, the behavioural influence staircase model developed by the FBI suggests that it is often necessary to establish rapport and trust with a suspect (i.e. develop a relationship with them), before moving on to behavioural influences, such as asking for information related to the crime (Ireland & Vecchi, 2009).

Finally, suspects in an identity frame would tend to focus on their own needs, values and beliefs (Wells & Brandon, 2019). For example, a suspect wanting to explore the reasons why an incident occurred, rather than providing informational details, would be communicating in an identity frame. Here, concepts such as ‘face’ which communicate respect and personal worth are important (Goffman, 1967). A common understanding is that a person’s identity can relate both to their social identity (e.g. race and gender; Tajfel & Turner, 1986) as well as their personal identity (e.g. personality traits and physical attributes; Petriglieri, 2011), with identity framed messages focusing on either one or both of them. Another way to understand communication that falls into an identity frame is that it may be used by speakers to attack (e.g. intimidation) and defend (e.g. blaming) a person’s self-reputation.

**Figure 1.**
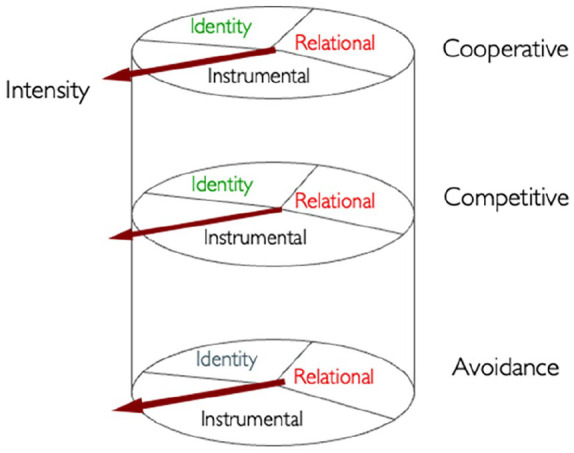
The cylinder model (taken from Taylor, 2002).

The three motivational frames were brought together into the cylinder model by Taylor (2002; see Figure 1). As Taylor and Donald (2007) showed, these frames not only dominate periods of dialogue as the interviewer and suspect move from issue to issue, but they also dominate whole interactions when the context dictates the focus of discussion.

## Relationship with related conversational theories

While the cylinder model and associated motivational frames were developed from crisis negotiation interactions (Taylor, 2002; Taylor & Donald, 2004), there are related theories and models in social psychology that have identified similar motivations. For instance, Laver and Hutcheson (1972) identified three different types of information communicated in interpersonal situations that largely aligned with the three motivational frames from the cylinder model: (a) cognitive information (similar to informational motivations), (b) interaction-management information (similar to relational motivations) and (c) indexical information (similar to identity motivations). Later work by Dunbar et al. (1997) showed that conversations in informal settings, such as in restaurants and bars, tended to revolve around relational (personal relationships) and identity topics (personal experiences), with a smaller fraction devoted to more instrumental topics (technical and work/academic discussions). Looking at a sample of English police interviews, Arnold (2021) found that suspects tended to focus their communication around instrumental, relational and identity motivational frames, providing further support for the relevance of such motivational frames to investigative interviews. Recently, the conversational circumplex model argued that people tend to have either informational or relational motivations in conversations (referred to as goals; Yeomans et al., 2022). In this model, informational goals can range from very high (e.g. brainstorming ideas or learning new things), to very low (e.g. fill time or avoid awkwardness), while relational goals may conversely range from low (e.g. assigning blame or claiming credit) to high (e.g. flattering or apologising).

Taken together, the large overlap between these different models and theories suggests that the three motivational frames from the cylinder model (instrumental, relational and identity) might capture a more universal description of interpersonal communication that would be relevant in a range of different situations, including investigative interviews. While the motivational frames are valuable for understanding a speaker’s motivations, it could be hypothesised that matching and aligning one’s own communication with the speaker is important for developing mutual understanding and cooperation.

## Alignment in communication

Theories about interpersonal interaction processes such as Communication Accommodation Theory (Giles & Ogay, 2007) and Interaction Alignment Model (Pickering & Garrod, 2004) propose that people tend to align their way of communicating to the extent that they want to associate and be liked by another person, and that doing so enables the development of a mutual understanding (i.e. common ground) and cooperation (Wachsmuth, 2013). For instance, research suggests that conversational transitions, such as initiations and terminations, are often carefully negotiated and coordinated (Stokoe, 2021). One measure of interpersonal coordination is language style matching, which provides a measure of how much two speakers coordinate their use of function words (e.g. articles and prepositions; Niederhoffer & Pennebaker, 2002).

Research on language style matching shows how similarity and synchrony in the type of language used underpin sensemaking. Richardson et al. (2019) examined how power and affiliation interacted with language style matching to predict success on a problem-solving task that required participants to make sense of each other. They found that task success was related to higher language style matching, but only for pairs with a symmetrical power relationship. In the forensic area, language style matching has been shown to be related to successful negotiation outcomes (Taylor & Thomas, 2008) and confessions in investigative interviewing situations (Richardson et al., 2014). Assuming motivational frame matching might work via similar mechanisms, it could be hypothesised that it would also have a positive influence on interaction outcomes.

Indeed, in relation to the cylinder model, previous research found that matching of motivational frames was associated with positive outcomes in crisis negotiations (Ormerod et al., 2008). Specifically, negotiations that ended peacefully saw a gradual increase in the length of motivational frame matching episodes between the perpetrator and the police negotiator, with the opposite trend observed in unsuccessful negotiations (Ormerod et al., 2008). Giebels et al. (2017) also found greater motivational frame matching between negotiators and perpetrators who shared their cultural background, suggesting that interpersonal sensemaking might be facilitated by having access to similar cultural experiences. Focusing on the positive outcomes of successful interpersonal sensemaking, an evaluation of the effectiveness of motivational frame matching for authentic investigative interviews (together with other interviewing techniques such as motivational interviewing and the cognitive interview) found a positive effect on suspects’ cooperation and information gain (Brandon et al., 2019).

Hence, it could be theorised that matching of motivational frames would lead to more positive perceptions of the interviewer and a greater willingness to cooperate with them. However, this has not been demonstrated experimentally, making it difficult to know whether matching leads to cooperation or is merely associated with it. By experimentally manipulating motivational frame matching, it is possible to closely examine its influence on interaction outcomes. Furthermore, it enables careful examination of how other variables (e.g. orientation) interact with motivational frame matching. Providing experimental evidence on the effectiveness of motivational frame matching is important. This is because it is taught to interviewers around the world and was included in the High Value Detainee Interrogation Group’s (2016) review of the science of interrogation. Establishing causal relationships has also been described as a hallmark of a cumulative science, of which psychology aspires to be (Eronen & Bringmann, 2021).

Consequently, in this study, we aim to present the first experimental evidence for a causal link between motivational frame matching and positive investigative interviewing outcomes. Based on the evidence above, we hypothesised that matching of motivational frames would lead to more positive interaction outcomes within an investigative interview (*H1*).

## Orientation to interaction

In addition to the motivational frames, the cylinder model also identifies three types of orientations people take to their interactions with others: cooperative, competitive and avoidant (Taylor, 2002; Taylor & Donald, 2004). Specifically, a suspect taking a cooperative orientation towards the interaction is seeking to engage, problem-solve and work toward a common objective. On the other hand, a suspect in a competitive orientation will show hostility and rigidity in thought, often giving one-sided justifications for their position and an unwillingness to consider alternatives. Finally, a suspect in an avoidant orientation will withdraw from an interaction, either deliberately or because of a light response. They may look away, making excuses or avoiding speaking altogether (Wells & Brandon, 2019). According to Taylor (2002), the orientations are orthogonal to the motivational frames such that people may focus on either of the three motivational frames while adopting a cooperative, competitive or avoidance orientation. For practical and theoretical reasons, in this article, we focus on the cooperative and competitive orientations to the interaction.

Studies of orientation in investigative interviews show overwhelmingly that orienting cooperatively to gather information is most likely to lead to cooperation from suspects (Meissner et al., 2015). Observing real investigative interviews, cooperative interview approaches have been demonstrated to lead to increased perceived rapport which, in turn, increased cooperation (Brandon et al., 2019), indicating the effectiveness of such approaches within an information gathering approach. Among American inmates themselves, perspective-taking and rapport were also mentioned as two of the most important factors for how the inmates wanted investigative interviewers to have treated them during their own interviews (Cleary & Bull, 2019). These findings highlight the beneficial outcomes of treating suspects in a positive and respectful way. Based on these findings, we hypothesised that a friendly and positive interaction between the investigative interviewer and suspect (i.e. a cooperative orientation) would lead to more positive interaction outcomes within an investigative interview (*H2*).

Although cooperation and competition can be conceptualised as being related to the goals and outcomes of an interaction, a typical approach in interpersonal interaction research is to see them as two different ways of relating to each other (e.g. this was termed affiliation by Keisler, 1983). This is exemplified in Leary’s interpersonal behavioural circumplex (1957), which identified behaviours as going from cooperative (e.g. seeking friendly feelings from others and complimenting others) to competitive behaviours (e.g. hostility and aggression). Leary’s circumplex model was later adapted by Alison and Alison (2017; 2020; ORBIT-model) for use within investigative interviews. Incidentally, the orientation dimension in Taylor’s (2002) cylinder model bears a strong resemblance to the cooperative-competitive dimension in both Leary’s and Alison and Alison’s models, indicating its comprehensiveness in explaining interpersonal orientation. Consequently, in the current study, cooperative and competitive orientations may be understood more as a way of relating to an interaction partner rather than a disagreement about objectives or goals (Deutsch, 2006).

As outlined by Sjöberg et al. (2023), the effect of matching motivations is dependent on the type of orientation a suspect takes. Specifically, it could be expected that motivational frame matching might lead to positive interaction outcomes when the suspect and interviewer are communicating in a cooperative way, but lead to worse interaction outcomes when the suspect and interviewer are communicating in a competitive way. There are three potential reasons for this. First, Levenson and Gottman (1983) found that arguing spouses matched their physiological arousal levels as their arguments increased in intensity, suggesting that matching may be an indication of involvement rather than being ubiquitously positive. More recently, Richardson et al. (2019) showed that language style matching was associated with task success for cooperative and symmetric dyads, while for competitive and symmetric dyads, language style matching was instead related to task failure. Finally, research has suggested that when people are competing with each other, matching (i.e. language style matching) actually leads to worse negotiation outcomes (Ireland & Henderson, 2014). These findings indicate that matching within interactions is not ubiquitously positive or negative, but rather, depends on the context of the interaction.

A way to resolve these supposedly contradictory observations about matching (that it can relate to both positive and negative interpersonal outcomes) would be to interpret matching and synchrony as signs of increased attention between interaction partners (Chartrand & Bargh, 1999; Ireland & Henderson, 2014). Hence, Ireland and Henderson (2014) suggested that matching is not inherently positive or negative, but rather, depends on the goals and motivations of the speakers. This goes in line with a social engagement theory of matching, which suggests that matching might be a sign that two people are actively focused on each other (Dalton et al., 2010; Ireland & Henderson, 2014). Another way to understand this is that motivational frame matching might lead to communication spiralling, with positive or negative outcomes dependent on the orientation taken towards the interaction (cooperative vs. competitive). Consistent with this, it was hypothesised that motivational frame matching would lead to more positive interaction outcomes within a cooperative orientation, but lead to less positive interaction outcomes within a competitive orientation (*H3*).

## A note on measuring interaction success

To study in detail the effects of motivational frame matching requires a sophisticated measurement of the outcomes desired within investigative interviews. Arguably, the most important outcome in an investigative interview is obtaining information that has some legal or operational value. For this to occur, suspects must first be willing to cooperate with the interviewer. Indeed, research suggests that cooperation is often necessary to obtain valuable information from suspects (Brandon et al., 2019). Hence, in the current study, both willingness to cooperate as well as providing information are used as two measures of positive interaction outcomes.

There are, however, other interaction outcomes that might be valuable for interviewers beyond information capture. One of the most studied is rapport, which we define as a positive working relationship between the suspect and interviewer (Abbe & Brandon, 2014). Critical components of rapport concern the ability to actively listen and empathise with a suspect (Alison & Alison, 2017), which, if done successfully, ensures suspects feel that they are being listened to and understood. In line with this, we measure feelings of being listened to and understood to tap into the interpersonal relationship between the suspect and interviewer.

Allowing a suspect to save face and treating them with respect might also be beneficial within an investigative interview for facilitating more positive interaction outcomes (Kleinman, 2006). For instance, Wells and Brandon (2019) described how a failure by the interviewer to fully respect a suspect led to a near termination of the interview. This is echoed in a study by Holmberg and Christianson (2002), who found that sexual offenders who did not feel respected by the investigative interviewer experienced feelings of alienation and a reduced likelihood of providing a confession. Similarly, Oxburgh and Ost (2011) argued that validating a suspect’s concerns would likely make them feel more accepted. To tap into these interaction outcomes, we measured suspects’ feelings of being treated fairly and with respect by the interviewer.

Finally, having a willingness to trust the interviewer might be crucial in order for them to start opening up about what happened (Brimbal et al., 2019). Balliet and Van Lange’s (2013) meta-analysis showed that trust was a particularly important predictor of cooperation in situations with large conflicts of interest (such as investigative interviews). We define interpersonal trust as an intention to accept vulnerability that is largely based on a positive expectation of how another person will act in the future (Rousseau et al., 1998). Working from this definition, Gillespie (2003, 2015) developed the behavioural trust inventory comprising two related constructs, (a) a willingness to rely on another person, and (b) a willingness to disclose sensitive information. The current research examined these two elements of a suspect’s trust.

In sum, the current research examined suspects’ willingness to provide information and cooperate with the interviewer, feelings of being understood, being treated fairly and with respect and intention to trust the interviewer, all as potential positive interaction outcomes.

## A note on the design of the current experiments

In both of the current experiments, participants could not actively respond to the interviewer at each round of the interview. Instead, the responses were pre-determined by the researchers. While an obvious drawback with this type of design is that participants could not decide how they wanted to respond to the interviewer at each interview round, there were also several key theoretical benefits of this experimental design. First, by controlling the motivational frames at each round of the interview, it was possible to experimentally manipulate fully matched versus fully nonmatched interactions. This is important as previous pilot studies found that participants tended not to stick with one motivational frame throughout the interview (i.e. they switched motivational frames several times throughout the course of the interview). This, in turn, would have made it difficult to obtain a clean interaction that was fully motivationally matched (in either an instrumental, relational or identity frame), or fully nonmatched. Second, having a fully matched interaction in either an instrumental, relational or identity motivational frame enabled analyses looking at the differences across the three frames (something that we return to in exploratory analyses available on the OSF). Finally, since the current study is, to the author’s knowledge, the first to have experimentally investigated the potential benefits of motivational frame matching on interaction outcomes, the decision was taken to keep the design as clear and simple as possible, in line with recent calls of seeing psychology as a cumulative science (Sharpe & Goghari, 2020) where simple, but robust, findings provide the foundations for more complex and elaborate studies.

## Experiment 1

The first experiment was designed to look at the effect of motivational frame matching on positive interaction outcomes. By experimentally manipulating motivational frame matching through a script-based investigative interview, it was possible to compare matched and nonmatched interactions against each other.

### Design

The current experiment employed a 2 (motivational frame matching vs. nonmatching) × 2 (cooperative vs. competive orientation) between participants experimental design. Participants were randomly allocated to the four experimental conditions.

### Method

#### Participants

##### Sample size determination and power analysis

An a priori power analysis suggested 359 participants were needed to achieve a power >0.9, with a small to medium effect size of *f* = 0.20 (which was the smallest effect of interest; Anvari & Lakens, 2021; this approximately converts into a Cohen’s d=0.40 ^1^ or a $\eta_{p}^{2}=.038$ ^2^) in the population (Cohen, 1988; Faul et al., 2007), at the nominal (.05) alpha error probability.^3^ The power was calculated to account for both main and interaction effects. Accordingly, we recruited 381 participants in return for financial compensation (£1.5; payment set in Prolific). Of these, four were excluded for failing to accurately respond to the attention check question. This left 377 participants for final analysis.

##### Sensitivity power analysis

With 377 participants, the first experiment would be sensitive to detect effect sizes of $f\geq 0.19;\mathrm{Cohen},s\;\;d\geq 0.38;\;\;\eta_{p}^{2}\geq .0348$ , that would be detectable with 90% power, and effect sizes of $f\geq 0.17;\;\;\mathrm{Cohen,s}\;\;d\geq 0.34;\;\eta_{p}^{2}\geq .028$ , that would be detectable with 80% power (both with alpha = .05). These relate both to the main and interaction effects in the subsequent statistical analyses.

In terms of demographics, 268 participants self-declared as women, 108 as men and 1 as other. They were aged between 18 and 76 years (*M* = 36.77, *SD* = 12.68). Most of them identified as White (*n* = 324), while the rest identified as either Asian (*n* = 18), Mixed (*n* = 9) or Black/African/Caribbean (*n* = 26). The study received ethical approval from a university in Northern England.

#### Materials

##### Investigative interview

The interview was a 5-round text-based interaction. Table 1 (cooperative) and Table 2 (competitive) provide the statements for each motivational frame for the matching interviews, while Tables 3 and 4 include the statements for the nonmatching interviews. In total, there were six matching and six nonmatching interviews, with half of them being cooperative and the other half being competitive.

**Table 1.** Conversational scripts for the cooperative orientation interaction with instrumental, relational and identity motivational frame responses for Experiment 1.

Interview round	Instrumental frame	Relational frame	Identity frame
Round 1-Interviewer	I: I am investigating the suspicion against you regarding the possession of illicit substances. Can you explain to us what happened?	I: I am investigating the suspicion against you regarding the possession of illicit substances. How are you feeling today?	I: I am investigating the suspicion against you regarding the possession of illicit substances. Why do you think you are here today?
Round 1-Suspect	S: Of course, it was just a normal day at work. Nothing special at all from what I can remember.	S: Thank you for asking. After all, it is nice to know that you care about how I feel. To be honest, I am not feeling great at the moment.	S: I don’t really know, I am not the kind of person who would do anything wrong. I always try to be a very honest person.
Round 2-Interviewer	I: Great, let’s start from the beginning and tell us what happened. What did you do in the morning?	I: Okay, I fully understand, it is completely normal to feel that way in your situation. We will do our best to help you through this.	I: Got it, you are an honest person, and an honest person would probably not do something that is alleged in this case.
Round 2-Suspect	S: Sure, I woke up and made breakfast around 8am. Then I drove to work and started my shift early.	S: That is really nice to hear that you are committed to helping me through this. Having a person like you care and listen to me makes a huge difference in the current situation.	S: Indeed, I do not think I have done something wrong. People who know me always say very good things about me and I would never do anything wrong.
Round 3-Interviewer	I: Did you notice anything unusual at that time? Any information might be of value to us.	I: I can reassure you that we are here to help you. We will try to listen as much as possible to what you are saying.	I: It sounds to me like you are a very trustworthy and reliable person. Such a person would probably not commit any kind of delinquent acts.
Round 3-Suspect	S: Everything was normal and I do not think anyone was there. I usually arrive before my co-workers.	S: That is nice to hear from you. It seems you are willing to listen to my story which means a lot to me and I really appreciate it.	S: Yeah right, I am really good at my job and people really respect me around here. I am afraid of what they would think of me if I was found guilty of something like this.
Round 4-Interviewer	I: Speed forward to later in the day, what was the last thing you did before returning home?	I: Indeed, I can reassure you that we will listen to your story. Helping you tell your side of what happened is very important for us.	I: It sounds like respect and admiration from other people are very important to you. I am sure we can find a way to uphold your admirable reputation.
Round 4-Suspect	S: I joked around a while with my co-workers before getting the keys to my car and then I went back home.	S: I am very grateful to you for letting me tell my side of the story. It would mean a lot to me if you also tried and support my story.	S: Yes, I do care about my reputation around here. It is important for me that people respect and appreciate me. Being convicted of something like this would get me into a lot of trouble.
Round 5-Interviewer	I: Thank you for providing this information, it is very valuable.	I: We will do our best to support your story. Thank you for speaking with me.	I: We will do our best to try and solve this case without harming your good reputation. Thank you for highlighting your concerns so clearly.
Round 5-Suspect	S: No worries, I hope it is helpful information.	S: No worries, thank you for listening to me.	S: No worries, thanks for honouring my concerns.

**Table 2.** Conversational scripts for the competitive orientation interaction with instrumental, relational and identity motivational frame responses for Experiment 1.

Interview round	Instrumental frame	Relational frame	Identity frame
Round 1-Interviewer	I: I am investigating the suspicion against you regarding the possession of illicit substances. Can you explain to us what happened?	I: I am investigating the suspicion against you regarding the possession of illicit substances. How are you feeling today?	I: I am investigating the suspicion against you regarding the possession of illicit substances. Why do you think you are here today?
Round 1-Suspect	S: I could tell you, but I do not see the value in telling you what happened. It would be better if you just told me why I am here.	S: What do you think? I am feeling a bit like crap to be honest. I am not sure what you all want from me.	S: I haven’t done anything wrong so I don’t really have a clue as to why I am here. This whole thing is a bit ridiculous if you ask me.
Round 2-Interviewer	I: Well, I already told you the charges against you. Now it is time for you to start speaking up and give me some information.	I: Well, I am not interested in listening to you whine about your emotions and how hurt you are, that is one thing that is for sure.	I: Well, since you are here talking with us, chances are you have done something wrong. There is nothing ridiculous at all about us or this investigation.
Round 2-Suspect	S: You keep saying that you told me the charges against me, but I am still not sure why I am here. It would be good if you could give me some insight on this.	S: That is a harsh thing to say to me. I feel like you special agents are all against me and I am pretty sure you will not believe a word I am saying.	S: I am sure there must be more important things for you to do than prosecute an innocent person like me. You should be embarrassed of yourself.
Round 3-Interviewer	I: Okay, I am telling you this for the last time. You are suspected of possession of illicit substances, and we want to know what happened.	I: Well, I would lie if I told you that we would believe every single word you are saying. Again, we are not here to be friends with you.	I: The only person here who is embarrassing is you. It does not surprise me that you do not have any close friends around here, the way you are behaving.
Round 3-Suspect	S: Well, do you have proof that I really have any involvement in this? If you do not provide me with evidence pertaining to my guilt, it is impossible for me to give you any information.	S: You guys clearly don’t like me at all. You seemed like good guys when I walked in here, but I was clearly wrong about you.	S: Don’t you dare tell me I have no friends around here. I am really good at my job, and people here like and respect me a lot.
Round 4-Interviewer	I: I cannot give you all of the evidence, that is classified information. What I can tell you is that it is about time you start speaking up and tell us what actually happened.	I: Yeah, we don’t really care about you. It would be impossible for us to care and empathise with every person we talk to. Especially, someone like you.	I: For some reason, I find that difficult to believe. The way you are behaving right now is not really typical of a respectful and honourable person.
Round 4-Suspect	S: If you cannot give me the evidence of my guilt, how do you expect me to provide you with any information? Tell me what you have on me first and then you might get your information.	S: That is not a very nice thing to say to me. But on the other hand, I am not too keen on helping you as well to be honest.	S: Who are you to judge me anyway? I cannot believe I am talking about respect and honour with a special agent.
Round 5-Interviewer	I: It sounds like you are not going to provide us with any information.	I: It sounds like you are not willing to help us with anything.	I: It sounds like you are more concerned about your reputation than speaking up.
Round 5-Suspect	S: Indeed, under the current circumstances, I will not give you any information.	S: Indeed, under the current circumstances, I do not want to help you at all.	S: Indeed, I do care about my reputation and I am not going to let you destroy it.

**Table 3.** Conversational scripts for the cooperative orientation interaction with nonmatching frame responses for Experiment 1.

Interview round	Nonmatching Interview 1	Nonmatching Interview 2	Nonmatching Interview 3
Round 1-Interviewer	I: I am investigating the suspicion against you regarding the possession of illicit substances. Can you explain to us what happened? (Ins)	I: I am investigating the suspicion against you regarding the possession of illicit substances. How are you feeling today? (Rel)	I: I am investigating the suspicion against you regarding the possession of illicit substances. Why do you think you are here today? (Ide)
Round 1-Suspect	S: Thank you for asking. After all, it is nice to know that you care about how I feel. To be honest, I am not feeling great at the moment. (Rel)	S: Of course, it was just a normal day at work. Nothing special at all from what I can remember. (Ins)	S: Of course, it was just a normal day at work. Nothing special at all from what I can remember. (Ins)
Round 2-Interviewer	I: Great, let’s start from the beginning and tell us what happened. What did you do in the morning? (Ins)	I: Okay I fully understand, it is completely normal to feel that way in your situation. We will do our best to help you through this. (Rel)	I: Okay I fully understand, it is completely normal to feel that way in your situation. We will do our best to help you through this. (Rel)
Round 2-Suspect	S: Indeed, I do not think I have done something wrong. People who know me always say very good things about me, and I would never do anything wrong. (Ide)	S: Indeed, I do not think I have done something wrong. People who know me always say very good things about me, and I would never do anything wrong. (Ide)	S: Indeed, I do not think I have done something wrong. People who know me always say very good things about me, and I would never do anything wrong. (Ide)
Round 3-Interviewer	I: Did you notice anything unusual at that time? Any information might be of value to us. (Ins)	I: Did you notice anything unusual at that time? Any information might be of value to us. (Ins)	I: I can reassure you that we are here to help you. We will try to listen as much as possible to what you are saying. (Rel)
Round 3-Suspect	S: That is nice to hear from you. It seems you are willing to listen to my story, which means a lot to me, and I really appreciate it. (Rel)	S: That is nice to hear from you. It seems you are willing to listen to my story, which means a lot to me, and I really appreciate it. (Rel)	S: Everything was normal and I do not think anyone was there. I usually arrive before my co-workers. (Ins)
Round 4-Interviewer	I: It sounds like respect and admiration from other people are very important to you. I am sure we can find a way to uphold your admirable reputation. (Ide)	I: It sounds like respect and admiration from other people are very important to you. I am sure we can find a way to uphold your admirable reputation. (Ide)	I: It sounds like respect and admiration from other people are very important to you. I am sure we can find a way to uphold your admirable reputation. (Ide)
Round 4-Suspect	S: I am very grateful to you for letting me tell my side of the story. It would mean a lot to me if you also tried and support my story. (Rel)	S: I am very grateful to you for letting me tell my side of the story. It would mean a lot to me if you also tried and support my story. (Rel)	S: I joked around a while with my co-workers before getting the keys to my car and then I went back home. (Ins)
Round 5-Interviewer	I: Thank you for providing this information, it is very valuable. (Ins)	I: Thank you for providing this information, it is very valuable. (Ins)	I: We will do our best to try and solve this case without harming your good reputation. Thank you for highlighting your concerns so clearly. (Ide)
Round 5-Suspect	S: No worries, thanks for honouring my concerns. (Ide)	S: No worries, thanks for honouring my concerns. (Ide)	S: No worries, hope it is helpful information. (Ins)

*Note*. Ins = instrumental frame, Rel = relational frame, Ide = identity frame.

**Table 4.** Conversational scripts for the competitive orientation interaction with nonmatching frame responses for Experiment 1.

Interview round	Nonmatching Interview 1	Nonmatching Interview 2	Nonmatching Interview 3
Round 1-Interviewer	I: I am investigating the suspicion against you regarding the possession of illicit substances. Can you explain to us what happened? (Ins)	I: I am investigating the suspicion against you regarding the possession of illicit substances. How are you feeling today? (Rel)	I: I am investigating the suspicion against you regarding the possession of illicit substances. Why do you think you are here today? (Ide)
Round 1-Suspect	S: What do you think? I am feeling a bit like crap to be honest. I am not sure what you all want from me. (Rel)	S: I could tell you, but I do not see the value in telling you what happened. It would be better if you just told me why I am here. (Ins)	S: I could tell you, but I do not see the value in telling you what happened. It would be better if you just told me why I am here. (Ins)
Round 2-Interviewer	I: Well, I already told you the charges against you. Now it is time for you to start speaking up and give me some information. (Ins)	I: Well, I am not interested in listening to you whine about your emotions and how hurt you are, that is one thing that is for sure. (Rel)	I: Well, I am not interested in listening to you whine about your emotions and how hurt you are, that is one thing that is for sure. (Rel)
Round 2-Suspect	S: I am sure there must be more important things for you to do than prosecute an innocent person like me. You should be embarrassed by yourself. (Ide)	S: I am sure there must be more important things for you to do than prosecute an innocent person like me. You should be embarrassed by yourself. (Ide)	S: I am sure there must be more important things for you to do than prosecute an innocent person like me. You should be embarrassed by yourself. (Ide)
Round 3-Interviewer	I: Okay, I am telling you this for the last time. You are suspected of possession of illicit substances, and we want to know what happened. (Ins)	I: Okay, I am telling you this for the last time. You are suspected of possession of illicit substances, and we want to know what happened. (Ins)	I: Well, I would lie if I told you that we would believe every single word you are saying. Again, we are not here to be friends with you. (Rel)
Round 3-Suspect	S: You guys clearly don’t like me at all. You seemed like good guys when I walked in here, but I was clearly wrong about you. (Rel)	S: You guys clearly don’t like me at all. You seemed like good guys when I walked in here, but I was clearly wrong about you. (Rel)	S: Well, do you have proof that I really have any involvement in this? If you do not provide me with evidence pertaining to my guilt, it is impossible for me to give you any information. (Ins)
Round 4-Interviewer	I: For some reason, I find that difficult to believe. The way you are behaving right now is not really typical of a respectful and honourable person. (Ide)	I: I cannot give you all of the evidence, that is classified information. What I can tell you is that it is about time you start speaking up and tell us what actually happened. (Ins)	I: For some reason, I find that difficult to believe. The way you are behaving right now is not really typical of a respectful and honourable person. (Ide)
Round 4-Suspect	S: That is not a very nice thing to say to me. But on the other hand, I am not too keen on helping you as well to be honest. (Rel)	S: That is not a very nice thing to say to me. But on the other hand, I am not too keen on helping you as well, to be honest. (Rel)	S: If you cannot give me the evidence of my guilt, how do you expect me to provide you with any information? Tell me what you have on me first, and then you might get your information. (Ins)
Round 5-Interviewer	I: It sounds like you are not going to provide us with any information. (Ins)	I: It sounds like you are not going to provide us with any information. (Ins)	I: It sounds like you are more concerned about your reputation than speaking up. (Ide)
Round 5-Suspect	S: Indeed, I do care about my reputation, and I am not going to let you destroy it. (Ide)	S: Indeed, I do care about my reputation, and I am not going to let you destroy it. (Ide)	S: Indeed, under the current circumstances, I will not give you any information. (Ins)

*Note*. Ins = instrumental frame, Rel = relational frame, Ide = identity frame.

In addition, the orientation of the interaction was manipulated as either cooperative (i.e. interviewer and suspect behaved in a relatively friendly manner) or competitive (i.e. they took a more hostile approach). For example, a cooperative statement made by the interviewer was ‘Thank you for providing this information, it is very valuable’. In contrast, a competitive statement was ‘Well, I already told you the charges against you. Now it is time for you to start speaking up and give me some information’.

##### Validity of the scripts

Before the experiment, we verified that the conversational encounters were perceived by experts to conform to one of the three motivational frames (instrumental, relational or identity) and the two orientations (cooperative or competitive). Consequently, three people familiar with the cylinder model assigned the interviewer questions and suspect responses into either instrumental, relational or identity motivational frames, as well as either the cooperative or competitive orientations. These individuals had extensive experience with the cylinder model as a result of working with and teaching about the model. Specifically, they were asked: ‘Please indicate the motivational frame and orientation that you consider each interaction belongs to’. The raters showed perfect (100%) agreement in correctly assigning both the motivational frames and the orientations on their first trial, suggesting that the encounters conformed well to their respective frame and orientation.

##### Language style matching

Since motivational frame matching could be hypothesised to influence language style matching, the conversational scripts from the matching and nonmatching conditions were compared in terms of their language style matching scores. There was no statistically significant difference in language style matching between the matching conditions (*M* = 0.60, *SD* = 0.044), and the nonmatching conditions (*M* = 0.56, *SD* = 0.032), *t*(9.1327) = 1.85, *p* = .097, *d* = 1.07, 95% CI [−0.31, 2.44]. While the effect size estimate was relatively large, it was nonsignificant, which limits its interpretability. In other words, the two conditions did not significantly differ when it came to language style matching, limiting the possibility that any observed findings would be due to language style matching.

##### Post-interview measures

After the participants had completed the interview, they answered questions relating to whether they, as suspects, would cooperate and provide information to the interviewer (i.e. instrumentally focused), whether they felt understood by the interviewer (i.e. relationally focused), how much they identified with the interviewer, and whether the interviewer had treated them with dignity and respect (i.e. identity focused). In addition, we also measured their intention to trust the interviewer.

##### Cooperating and providing information to the interviewer

Participants were asked whether they would be willing to cooperative with the interviewer and, if they had information about the crime, how likely they would be to give this information to the interviewer.^4^ These single-item measures were answered on a 7-point Likert scale anchored by 1 (*Not at all willing*) to 7 (*Completely willing*). Since these were single-item measures, they did not have a Cronbach’s α -score associated with them.

##### Feeling understood by the interviewer

This measure focused on the participants’ feelings about the interviewer and whether they felt listened to and understood by the interviewer. An example item was ‘I felt understood by the interviewer’. In total, there were three items in this scale, and they were all answered on a 7-point Likert scale anchored by 1 (*Disagree strongly*) to 7 (*Agree strongly*). The scale demonstrated excellent Cronbach’s α = .96.

##### Perceptions of being treated with respect

To tap into participants’ identity-focused concerns, two sets of questions asked whether they felt the interviewer had treated them with dignity and respect. An example item was ‘I felt the interviewer treated me with dignity’. As before, this scale was answered on a 7-point Likert scale anchored by 1 (*Disagree strongly*) to 7 (*Agree strongly*).

##### Inclusion of the other in the self scale

We used the ‘inclusion of other in the self’ scale (Aron et al., 1992) to measure interpersonal closeness with the interviewer. This scale presents pairs of circles with varying degrees of overlap and asks a participant to select the pair of circles that best describes their relationship with the interviewer. As this scale tapped into somewhat similar concerns as the previous two identity-focused questions (Pearson’sr>.6) , they were merged into a single identity scale. This scale demonstrated very good internal reliability (Cronbach’s α = .89).

##### Intention to trust

We used items from Gillespie’s (2003, 2015) behavioural trust inventory to tap into participants’ intention to trust the interviewer. The items included both a willingness to disclose feelings to the interviewer (e.g. ‘How willing are you to share your personal feelings with your interviewer?’) as well as a willingness to rely on the interviewer (e.g. ‘How willing are you to rely on your interviewer’s task-related skills and abilities?’). These items were answered on a 7-point Likert scale from 1 (*Not at all willing*) to 5 (*Completely willing*). This measure showed excellent internal consistency (Cronbach’s α = .97).

##### Demographic questions

Before the termination of the study, participants answered questions about their age, gender, ethnicity and country of residence.

#### Procedure

Participants on the Prolific website who self-selected for participation were given information about the study and provided informed consent. They were then sent to the Qualtrics experimental platform, where the study took place. Prolific is an online platform that connects researchers with potential research participants, while Qualtrics is a powerful online survey and experimental platform. Participants were then given background information about the crime the suspect was accused of. Specifically, the background information stated:

After a routine drug test at work, you (the suspect) tested positive for use of illicit substances. As a result of this, you have been referred to the police to be questioned by a law enforcement officer about what happened. The interviewer’s goal is to determine whether or not you used any unlawful substances. You will observe the interaction between the interviewer and the suspect. During the interaction, please imagine being in the suspect’s shoes. That is, picture yourself as being the suspect and envision how you would feel if you were in their situation.

As they observed a short interaction between the interviewer and the suspect, they were asked to imagine being in the suspect’s shoes and think about how they would feel if they were in the same situation. Depending on the condition, the interaction was either completely matched (instrumental, relational or identity motivational frames) or randomly nonmatched. In addition, the interaction was either cooperative or competitive. After the interview, participants answered the post-measures and were debriefed. Two hundred and ninety participants were randomly assigned to read a matching interview (143 cooperative interviews; 147 competitive interviews), while 87 participants were randomly assigned to read a nonmatching interview (46 cooperative interviews; 41 competitive interviews).^5^

#### Open science and disclosure statement

The hypotheses for this study were pre-registered on the Open Science Framework (anonymised link: https://osf.io/6dpny/?view_only=8db341d4271f43d184252b386ac6daac). The data and R-scripts used to analyse the data are also available online. All studies, measures, manipulations and data/participant exclusions are reported in the manuscript or its online Supplemental Material.

### Results

Before carrying out the statistical analyses, outliers were removed and replaced with the next highest/lowest score in line with Tabachnick and Fidell (2013). As a form of sensitivity analysis (Thabane et al., 2013), the removal of outliers did not change the direction or significance of the statistical tests. Table 5 shows the descriptive statistics for the outcome variables.

**Table 5.** Means (*SD*) for frame (matching vs. nonmatching) and orientation (cooperative vs. competitive) across all the dependent variables.

Dependent variables	Cooperative		Competitive	
	Matching	Nonmatching	Matching	Nonmatching
Willingness to cooperate with interviewer	**6.09 (1.24)**	**5.61 (1.48)** *	**2.47 (1.42)**	**3.05 (1.48)** *
Willingness to provide information	5.44 (1.52)	5.28 (1.56)	**2.84 (1.63)**	**3.49 (1.68)** *
Feeling understood	**5.77 (1.52)**	**4.59 (1.56)** ***	1.79 (1.63)	2.07 (1.68)
Identification with interviewer	**5.37 (1.03)**	**4.85 (1.14)** **	**1.61 (0.80)**	**1.91 (0.82)** *
Trust intention	**4.94 (1.28)**	**4.45 (1.47)** **	2.01 (0.87)	2.39 (1.01)

*Note*. Pairs in bold indicate a statistically significant difference *p < .05, **p < .01, ***p < .001.

In order to investigate the effect of matching and orientation on the outcome variables, a multivariate analysis of variance was initially carried out. This test was entered as 4 (frame: instrumental vs. relational vs. identity vs. nonmatched) × 2 (orientation: cooperative vs. competitive) between subjects MANOVA. Initial analyses suggested that all the outcome variables correlated relatively highly with each other (r>.7) , which is advised for MANOVA (Pallant, 2005). While the Box’s *M*-test for the homogeneity of covariance matrices was significant, $\chi^{2}\left(105\right)=262.48,p<.001$ , it has been argued that for large samples, such as in this study, the Box’s *M*-test tends to be too severe (Tabachnick & Fidell, 2013). Nevertheless, the Pillai’s Trace statistic was used throughout the analyses as it is often the most robust (Pallant, 2005; Tabachnick & Fidell, 2013).

For the combined dependent variables, there was a significant effect of frame, F(3,369)=4.52,p<.001, ${\mathrm{Pillai}}^{'}s\;\;\mathrm{Trace}=0.17,\;\;\eta_{p}^{2}=.03,\;\;95\%\;\;\mathrm{CI}\;[0.01,\;\;1.00]$ a significant effect of orientation, F(3,369)=265.22, F(3,369)=265.22, $p<.001,\;\;{\mathrm{Pillai}}^{'}s\;\mathrm{Trace}=0.78,\;\;\eta_{p}^{2}=.65,\;\;[0.61,\;\;1.00]$ and a significant interaction between frame and orientation, $F\left(3,\;\;369\right)=5.21,\;\;p<.001,\;\;{\mathrm{Pillai}}^{'}s\;\;\mathrm{Trace}=0.20,\;\;\eta_{p}^{2}=.04,$ [0.01,1.00] . To break down these differences, separate analysis of variance tests were conducted for each dependent variable. In line with our pre-registration, these analyses focused on the matching and nonmatching conditions. Individual-level analyses comparing each of the three frames with each other are available on the OSF (https://osf.io/6dpny/?view_only=8db341d4271f43d184252b386ac6daac).

#### Willingness to cooperate and provide information

##### Willingness to cooperate

There was a significant main effect of orientation, F(1,369)=154.62,p<.001 , $\;\eta_{2}^{p}=.30,\;\;95\%\;\;\mathrm{CI}\;[0.23,1.00]$ , as well as of motivational frame, $F\left(3,\;\;369\right)=2.80,\;\;p=.04,\;\;\eta_{2}^{p}=.02,\;\;[0.00,\;\;1.00]$ , on willingness to cooperate with the interviewer. Moreover, there was a significant interaction effect between frame and orientation, F(3,369)=3.33,p=.020, $\eta_{2}^{p}=.03,$ 9[0.00,1.00] .

As predicted, participants were more willing to cooperate in the cooperative (*M* = 5.69, *SD* = 1.30) compared to the competitive interaction (*M* = 2.79, *SD* = 1.41; β = $1.67,\;\;t=23.73,\;\;p<.001,\;\;\eta_{2}^{p}=.61,\;\;95\%\;\;\mathrm{CI}\;[0.56,\;\;1.00]$ ), supporting *H2* (a friendly and positive interaction between the investigative interviewer and suspect would lead to more positive interaction outcomes).

**Figure 2.**
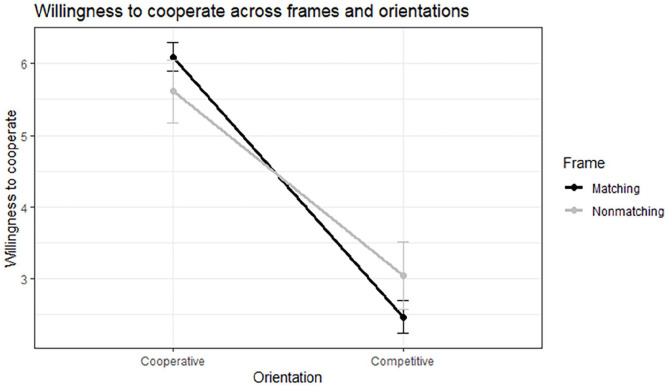
Interaction between frame (matching/nonmatching) and orientation (cooperative/competitive) on willingness to cooperate with the interviewer. Error bars represent standard errors.

Planned simple effects tests^6^ showed that, for the cooperative interaction, participants were more willing to cooperate in the matching (*M* = 6.09, *SD* = 1.24) versus nonmatching interaction (*M* = 5.61, *SD* = 1.48; β=.12,t=2.07, p=.0395,d=0.37,95%CI[0.04,0.70]) , providing support for *H1* (matching of motivational frames would lead to more positive interaction outcomes). However, for the competitive interaction, the opposite pattern was observed, with participants being more willing to cooperate with the interrogator in the nonmatching (*M* = 3.05, *SD* = 1.48) compared to the matching condition (*M* = 2.47, *SD* = 1.42; β=−.14, t=−2.37,p=.0186, d=0.41,[0.06,0.75] ), lending support for *H3* (motivational frame matching would lead to more positive interaction outcomes for a cooperative orientation, but lead to less positive interaction outcomes for a competitive orientation). This interaction is displayed in Figure 2.

##### Willingness to provide information

There was no main effect of frame, $F\left(3,\;\;369\right)=0.15,\;\;p=.93,\;\;\eta_{2}^{p}=.00,$ 95%CI[0.00,1.00] , but a main effect of orientation, $F\left(1,\;\;369\right)=80.54,\;\;p<.001,\;\;\eta_{2}^{p}=.18,\;[0.12,\;\;1.00]$ , as well as a significant frame by orientation interaction, $F\left(3,\;\;369\right)=3.64,\;\;p=.013,\;\;\eta_{2}^{p}=.03,\;\;[0.00,1.00]\;$ , on the willingness to provide information to the interviewer.

As predicted, participants were more willing to provide information in the cooperative (*M* = 5.40, *SD* = 1.53) compared to the competitive interaction (*M* = 2.98, *SD* = 1.66; $\beta =1.19,\;t=14.69,\;p<.001,\;\;\eta_{2}^{p}=.37,\;\;95\%\;\mathrm{CI}\;[0.31,\;\;1.00]$ ), lending support for *H2* (a friendly and positive interaction between the investigative interviewer and suspect would lead to more positive interaction outcomes).

Using simple effects tests (see note 6), within a cooperative interaction, there was no significant difference in willingness to provide information between the matching (*M* = 5.44, *SD* = 1.52) and nonmatching condition (*M* = 5.28, *SD* = 1.56; β=.039,t=0.58,p=.562,d=0.10,95%CI [−0.23,0.44] ), offering no support of *H1* (matching of motivational frames would lead to more positive interaction outcomes). However, within a competitive interaction, motivational frame matching did lead to a lower willingness to provide information (*M* = 2.84, *SD* = 1.63) than when the interaction was motivationally nonmatched (*M* = 3.49, *SD* = 1.68; β=−.16,t=−2.26,p=.025,d=0.40, [0.05,0.74]e ). This gives partial support for *H3* (motivational frame matching would lead to more positive interaction outcomes for a cooperative orientation but lead to less positive interaction outcomes for a competitive orientation).

#### Feeling understood

In terms of feelings of being understood by the interviewer, there was a significant main effect of frame,^7^ $F\left(3,\;\;369\right)=10.41,\;\;p<.001,\;\;\eta_{2}^{p}=.08,\;95\%\;\;$ CI[0.04,1.00] , a main effect of orientation, F(1,369) $=334.89,\;\;p<.001,\;\;\eta_{2}^{p}=.48,\;\;[0.42,\;\;1.00]$ , as well as a significant interaction effect between them, F(3,369) $=14.073,\;\;p<.001,\;\;\eta_{2}^{p}=.10,[0.05,\;1.00]$ .

As predicted, participants felt significantly more understood by the interviewer in the cooperative (*M* = 5.46, *SD* = 1.48) compared to the competitive interaction (*M* = 1.90, *SD* = 1.12; β=.49,t=29.80,p<.001,d=2.93, 95%CI[2.64,3.22] ), supporting *H2* (a friendly and positive interaction between the investigative interviewer and suspect would lead to more positive interaction outcomes).

Furthermore, simple effect tests (see note 6) showed that, for a cooperative interaction, participants felt more understood by the interviewer in the matching (*M* = 5.77, *SD* = 1.52) compared to the nonmatching condition (*M* = 4.59, *SD* = 1.56; β=.076,t=5.59,p<.001,d=0.89, 95%CI[0.54,1.23] ), while for the competitive interaction, there was no difference in feelings of being understood between the matching (*M* = 1.79, *SD* = 1.63) versus the nonmatching condition (*M* = 2.07, *SD* = 1.68; β=−.027, t=−1.93,p=.054,d=0.28,[−0.06,0.63] ), partially supporting *H1* (matching of motivational frames would lead to more positive interaction outcomes) and *H3* (motivational frame matching would lead to more positive interaction outcomes for a cooperative orientation but lead to less positive interaction outcomes for a competitive orientation).

#### Identification with the interviewer

Regarding the tendency to identify with the interviewer, there was a significant main effect of both frame (see note 7) β=−.027, $=6.033,\;p<.001,\eta_{2}^{p}=.053,95\%\;\mathrm{CI}\;\;[0.01,1.00]$ , and orientation $F\left(1,369\right)=499.050,p<.001,\eta_{2}^{p}=.57,[0.53,1.00]$ , as well as a significant interaction effect, F(3,369)=25.66, $p<.001,\;\;\eta_{2}^{p}=.17,\;\;[0.11,\;\;1.00]$ .

In line with expectations, participants identified more with the interviewer in the cooperative (*M* = 5.24, *SD* = 1.08) compared to the competitive interaction (*M* = 1.67, *SD* = 0.81; β=.50,t=40.63,p<.001,d=3.75, 95%CI[3.41,4.08] ), supporting *H2* (a friendly and positive interaction between the investigative interviewer and suspect would lead to more positive interaction outcomes).

**Figure 3.**
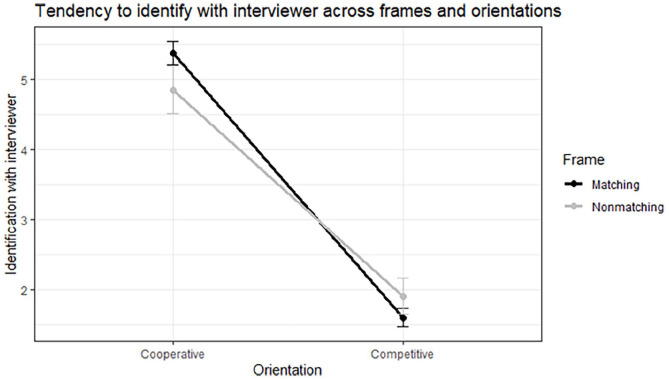
Interaction between frame (matching/nonmatching) and orientation (cooperative/competitive) on the tendency to identify with the interviewer.

Moving on to the matching hypotheses, simple effect tests (see note 6) demonstrated that, for a cooperative interaction, participants identified more with the interviewer in the matching (*M* = 5.37, *SD* = 1.03) compared to the nonmatching condition (*M* = 4.85, *SD* = 1.14; β=.030, t=2.92,p=.0038,d=0.50,95%CI[0.16,0.83] ), while for a competitive interaction, participants identified more with the interviewer in the nonmatching (*M* = 1.91, *SD* = 0.82) versus the matching condition (*M* = 1.61, *SD* = 0.80; β=−.027,t=−2.55,p=.011,d=0.38, [0.03,0.73] ), partially supporting *H1* (matching of motivational frames would lead to more positive interaction outcomes), and supporting *H3* (motivational frame matching would lead to more positive interaction outcomes for a cooperative orientation but lead to less positive interaction outcomes for a competitive orientation). This interaction is displayed in Figure 3.

#### Interviewer trust

When it came to the intention to trust the interviewer, there was a significant main effect of frame $F\left(3,\;\;369\right)=4.92,\;\;p=.0023,\;\;\eta_{2}^{p}=.04,\;\;95\%\;\mathrm{CI}\;[0.01,\;\;1.00]$ , a main effect of orientation F(1,369)=169.19, p<.001, $\eta_{2}^{p}=.31,\;\;[0.25,\;\;1.00]$ , as well as a significant interaction effect between frame and orientation, F(3,369)=6.78, $p<.001,\;\;\eta_{2}^{p}=.05,\;\;[0.02,\;\;1.00]$ .

As expected, participants trusted the interviewer more in the cooperative (*M* = 4.82, *SD* = 1.34) compared to the competitive interaction (*M* = 2.09, *SD* = 0.91; β=1.35, t=23.21,p<.001,d=2.37,95%CI[2.11,2.64] ), in line with *H2* (a friendly and positive interaction between the investigative interviewer and suspect would lead to more positive interaction outcomes).

For the matching hypotheses, simple effect tests (see note 6) demonstrated that, for a cooperative interaction, participants trusted the interviewer more in the matching (*M* = 4.94, *SD* = 1.28) compared to the nonmatching condition (*M* = 4.45, *SD* = 1.47; β=.12,t=2.49,p=.013,d=0.36, 95%CI[0.03,0.70] ), while for a competitive interaction, there was no significant difference between the matching (*M* = 2.01, *SD* = 0.87) and nonmatching conditions (*M* = 2.39, *SD* = 1.02; β=−.093,t=−1.87,p=.062, d=0.42, [0.07,0.77] ), partially supporting *H1* (matching of motivational frames would lead to more positive interaction outcomes), and *H3* (motivational frame matching would lead to more positive interaction outcomes for a cooperative orientation but lead to less positive interaction outcomes for a competitive orientation).

### Discussion Experiment 1

The aim of the first experiment was to investigate a potential causal link between motivational frame matching and positive interaction outcomes within an investigative interview. While motivational frame matching did not lead to a higher willingness to provide information within a cooperative interaction, it did lead to a higher willingness to cooperate with the interviewer, greater feelings of being understood, trust and identification with the interviewer. Conversely, within a competitive interaction, motivational frame matching led to less willingness to cooperate, provide information and trust the interviewer. As expected, interacting with a friendly and positive (i.e. cooperative) interviewer led participants to be more willing to cooperate, provide information, feel understood, identify and trust the interviewer. These results provide the first evidence of a causal link between motivational frame matching and positive interaction outcomes, such as willingness to cooperate, in investigative interviews. However, the results also point to the moderating role of orientation in influencing the effect of motivational frame matching.

## Experiment 2

Although the first experiment found a significant effect of motivational frame matching on positive interaction outcomes, a potential limitation might have been that the script-based interview was somewhat abstract and hypothetical. Hence, to increase the realism of the experiment and to make it more closely resemble an authentic interview situation, Experiment 2 had participants watch a video of a simulated investigative interview. Similar to Experiment 1, the aim of Experiment 2 was to investigate the role of motivational frame matching on positive interaction outcomes. These changes, from a script based to a video-based version of the experiment, constituted the only modification from the first to the second experiment.

### Method

#### Participants

##### Sample size determination and power analysis

An a priori power analysis suggested that 359 participants were needed to reach a power >0.9, provided a small to medium effect size of (*f* = 0.20; which was the smallest effect of interest; Anvari & Lakens, 2021; this approximately converts into a Cohen,sd=0.40 (see note 1) or a $\eta_{p}^{2}=.038$ (see note 2)) in the population (Cohen, 1988; Faul et al., 2007), at the nominal (.05) alpha error probability (see note 3). The power was calculated to account for both main and interaction effects. Hence for this experiment, we recruited 408 participants in return for financial compensation (£.85; payment set in Prolific). Before data analysis, we removed nine participants from the dataset as they failed to accurately answer the attention check question. This left 399 participants for final data analysis. Of the remaining participants, 259 self-declared as women, 135 as men and 5 as other. Their ages ranged from 18 to 80 years (*M* = 38.49, *SD* = 13.4). Most of them identified as White (*n* = 301), while the other participants identified as either Asian (*n* = 26), Mixed (*n* = 10), Black/African/Caribbean (*n* = 56) or other (*n* = 6). The study received ethical approval from a university in Northern England.

##### Sensitivity power analysis

With 399 participants, the second experiment would be sensitive to detect effect sizes of $f\geq 0.189;\;\;\mathrm{Cohen’s}\;d\geq 0.378;\;\;\eta_{p}^{2}\geq .034$ , that would be detectable with 90% power, and effect sizes of $f\geq 0.166;\;\;\mathrm{Cohen’s}\;d\geq 0.33;\;\;\eta_{p}^{2}\geq .0268$ , that would be detectable with 80% power (both with alpha = .05). These relate both to the main and interaction effects in the subsequent statistical analyses.

#### Materials

##### Interview video

A simulation of an investigative interview was constructed with the help of two confederates, one acting as the suspect and the other as the interviewer. The Confederates were psychology students with previous acting experience. The interviewer asked five questions to the suspect, who then answered each question. Following on from Experiment 1, the questions and answers were either of the same motivational frame (i.e. matched instrumental, matched relational or matched identity) or randomly nonmatched motivational frames. These were combined with either an interaction where the suspect and interviewer took a cooperative orientation towards the interaction, or one where they instead took a competitive orientation. The interview videos are available on the open science framework (https://osf.io/6dpny/?view_only=8db341d4271f43d184252b386ac6daac). Similarly to the first experiment, there were six matching and six nonmatching interviews, with half of them being cooperative and the other half being competitive.

##### Validity of the interview videos

To ensure that the interview videos accurately conformed to the matching (instrumental, relational and identity motivational frames) and orientation conditions (cooperative and competitive), two independent raters familiar with the cylinder model, but unfamiliar with the study hypotheses, judged each of the scripts in terms of the interactants’ motivational frame and orientation. As before, these individuals had extensive experience with the cylinder model as a result of working with and teaching about the model. Specifically, they were asked: ‘Please indicate the motivational frame and orientation that you consider each interaction belongs to’. Their agreement was perfect (100%) in the first rating round and conformed to the study design, suggesting that the scripts used in the videos corresponded well to their experimental conditions.

##### Language style matching

Similar to the first experiment, the conversational scripts from the matching and nonmatching conditions were compared to explore whether they differed in terms of their language style matching scores. Again, there was no statistically significant difference in language style matching scores between the matching (*M* = 0.59, *SD* = 0.032), and nonmatching conditions (*M* = 0.58, *SD* = 0.070), *t*(6.9702) = 0.45, *p* = .67, *d* = 0.26, 95% CI [−1.03, 1.55] suggesting that the two conditions were similar in terms of language style matching.

##### Post-interview measures

After the participants watched the interview, they answered the same questions as Experiment 1. The internal reliability was again very good for the scale measures: feeling understood (Cronbach,sα=.94) , the tendency to identify with the interviewer (Cronbach,sα=.83) and intention to trust the interviewer (Cronbach,sα=.96). As before, willingness to cooperate and willingness to provide information were single-item measures and therefore did not have an associated α -score.

#### Procedure

Participants who volunteered to take part in the Prolific website were given information about the study and provided informed consent. They were then sent to the Qualtrics experimental platform, where the study took place. Prolific is an online platform that connects researchers with potential research participants, while Qualtrics is a powerful online survey and experimental platform. They were then given some background information about the crime the suspect was accused of. As they observed the 5-round video interaction between the interviewer and the suspect, they were asked to imagine taking the suspect’s perspective and to think about how they would feel if they were in the same situation. Depending on the condition, the interaction was either completely matched (instrumental, relational or identity motivational frames) or randomly nonmatched. In addition, the interaction was either cooperative or competitive. Two hundred participants were randomly assigned to watch a matching interview (102 cooperative interviews; 98 competitive interviews), while 199 participants were randomly assigned to watch a nonmatching interview (99 cooperative interviews; 100 competitive interviews). After the interview, participants answered the post-measures and were debriefed.

### Results

Consistent with Experiment 1, outliers were removed and replaced with the next highest/lowest score in line with Tabachnick and Fidell (2013). Conducted as a form of sensitivity analysis (Thabane et al., 2013), the removal of outliers did not change the direction or significance of the statistical tests. Descriptive statistics for the five outcome variables across frames and orientations are displayed in Table 6.

**Table 6.** Means (*SD*) for frame (matching vs. nonmatching) and orientation (cooperative vs. competitive) across all the dependent variables.

Dependent variables	Cooperative		Competitive	
	Matching	Nonmatching	Matching	Nonmatching
Willingness to cooperate with interviewer	**5.88 (0.96)**	**5.47 (1.08)** *	**2.52 (1.16)**	**3.23 (1.69)** ***
Willingness to provide information	5.57 (1.27)	5.20 (1.60)	3.39 (1.70)	3.72 (1.73)
Feeling understood	**5.73 (0.98)**	**5.08 (1.37)** ***	2.49 (1.38)	2.58 (1.41)
Identification with interviewer	5.21 (1.01)	4.96 (1.02)	**2.42 (1.22)**	**2.77 (1.40)** *
Trust intention	4.75 (1.14)	4.44 (1.37)	2.34 (1.12)	2.69 (1.29)

*Note*. Pairs in bold indicate a significant difference *p < .05, ***p < .001.

Similar to Experiment 1, to investigate the effect of matching and orientation on the outcome variables, a multivariate analysis of variance was performed. This test was entered as a 4 (frame: instrumental vs. relational vs. identity vs. nonmatched) × 2 (orientation: cooperative vs. competitive) between-subjects MANOVA. Preparatory analyses indicated that all the outcome variables correlated highly with each other (r>.5) , which is recommended for MANOVA (Pallant, 2005). As for the previous experiment, the Box’s *M*-test for the homogeneity of covariance matrices was significant, $\chi^{2}\left(45\right)=160.07,\;\;p<.001$ . However, for large samples, such as in this study, the Box’s *M*-test is often overly strict (Tabachnick & Fidell, 2013). Still, the Pillai’s Trace statistic was used throughout the analyses as it is usually the most robust (Pallant, 2005; Tabachnick & Fidell, 2013).

For the combined dependent variables, there was a significant effect of frame, F(3,391)=2.53,p=.0011, Pillai,sTrace=0.094, $\eta_{p}^{2}=.03,\;\;95\%\;\mathrm{CI}\;[0.01,\;1.00]$ , a significant effect of orientation, F(1,391)=146.063,p<.001, $\mathrm{Pillai,s}\;\mathrm{Trace}=0.65,\;\eta_{p}^{2}=.65,\;[0.61,\;1.00]$ and a significant interaction between frame and orientation, F(3,391)=3.29, $p<.001,\;\;\mathrm{Pillai,s}\;\mathrm{Trace}=0.12,\;\eta_{p}^{2}=.04,\;[0.01,\;1.00]$ . To break down these differences, separate analysis of variance tests were conducted for each dependent variable. As per our pre-registration, the below analyses focused on comparing the matching and nonmatching conditions. Individual level analyses comparing each of the three frames with each other are available on the OSF (https://osf.io/6dpny/?view_only=8db341d4271f43d184252b386ac6daac).

#### Willingness to cooperate and provide information

##### Willingness to cooperate with the interviewer

There was a significant main effect of both frames $F\left(3,\;391\right)=6.46,\;\;\;p<.001,\;\;\;\eta_{2}^{p}=\;.02,\;\;95\%\;\mathrm{CI}\;[0.00,\;1.00],$ and orientation $F\left(1,\;\;391\right)=92.76,\;\;p<.001,\;\;\eta_{2}^{p}=.05,\;$ [0.02,1.00] , as well as a significant interaction effect on the willingness to cooperate with the interviewer $F\left(3,\;\;391\right)=8.039,\;\;p<.001,\;\;\eta_{2}^{p}=.02,\;[0.00,\;1.00]$ .

As predicted, participants were more willing to cooperate with the interviewer when the interaction was cooperative (*M* = 5.68, *SD* = 1.04) rather than competitive in nature (*M* = 2.88, *SD* = 1.49; β=1.40,t=22.32,p<.001, d=2.18,95%CI[1.93,2.43] ), giving support for *H2* (a friendly and positive interaction between the investigative interviewer and suspect would lead to more positive interaction outcomes).

**Figure 4.**
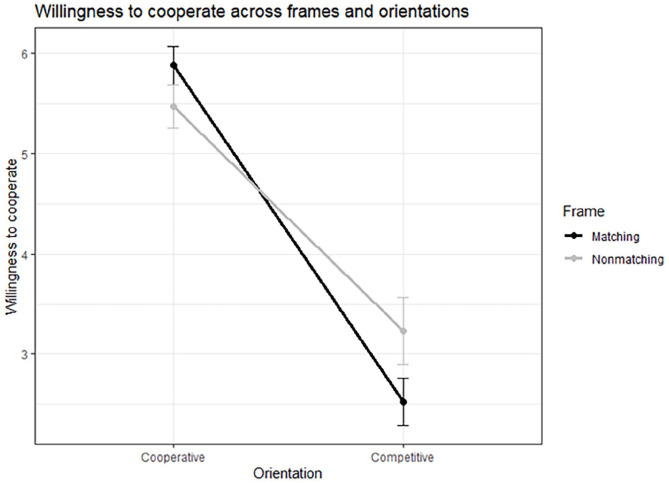
Interaction between frame Interaction between frame (matching/nonmatching) and orientation (cooperative/competitive) on willingness to cooperate with the interviewer. Error bars represent standard errors.

The significant interaction was followed-up with simple effects tests (see note 6). For the cooperative interaction, motivational frame matching led to significantly higher willingness to cooperate (*M* = 5.88, *SD* = 0.96) compared to a nonmatched interaction (*M* = 5.47, *SD* = 1.08, β=0.20,t=2.30,p=.022,d=0.40,95%CI[0.12,0.68]) , in line with *H1* (matching of motivational frames would lead to more positive interaction outcomes). Conversely, for the competitive interaction, motivational frame matching led to significantly less willingness to cooperate with the interviewer (*M* = 2.52, *SD* = 1.16) compared to the nonmatching interaction (*M* = 3.23, *SD* = 1.69, β=−0.35, t=−3.98,p<.001,d=0.49,[0.20,0.77]) . This gives support for *H3* (motivational frame matching would lead to more positive interaction outcomes for a cooperative orientation but less positive interaction outcomes for a competitive orientation). The interaction is displayed in Figure 4.

##### Willingness to provide information to the interviewer

While there was no main effect of frame F(3,391)= $2.46,\;\;p=.07,\;\;\eta_{2}^{p}=.02,\;\;95\%\;\;\mathrm{CI}\;[0.00,\;1.00]$ , there was a main effect of orientation F(1,391)=22.36, $\;p<.001,\;\;\eta_{2}^{p}=.05,\;\;[0.02,\;\;1.00]$ , with participants being more willing to provide information to the interviewer in the cooperative (*M* = 5.39, *SD* = 1.45) compared to the competitive orientation (*M* = 3.56, *SD* = 1.72; β=.92,t=11.55,p<.001,d=1.15,[0.94,1.37] ), again supporting *H2* (a friendly and positive interaction between the investigative interviewer and suspect would lead to more positive interaction outcomes). There was no interaction effect between frame and orientation, $F\left(3,\;\;391\right)=2.17,\;\;p=.092,\;\;\eta_{2}^{p}=.02,\;\;[0.00,\;\;1.00]$ .

#### Feeling understood

There was no significant main effect of frame, $F\left(3,\;\;391\right)=1.30,\;\;p=.27,\;\;\eta_{2}^{p}=.00,\;\;95\%$ CI[0.00,1.00] , but a significant effect of orientation, $F\left(1,\;\;391\right)=117.45,\;\;p<.001,\;\;\eta_{2}^{p}=.23,\;\;[0.17,\;\;1.00]$ , as well as a significant interaction effect, F(1,391)=22.36, $3.048,\;\;p=.029,\;\;\eta_{2}^{p}=.02,\;\;[0.00,\;\;1.00]$ , for feelings of being understood by the interviewer.

As predicted, participants felt more understood by the interviewer in the cooperative (*M* = 5.41, *SD* = 1.23) versus the competitive interaction (*M* = 2.53, *SD* = 1.39; β=1.44, t=22.16,p<.001,d=2.19,95%CI[1.94,2.44] ), providing support for *H2* (a friendly and positive interaction between the investigative interviewer and suspect would lead to more positive interaction outcomes).

Simple effect tests (see note 6) demonstrated that, within the cooperative interaction, participants felt more understood by the interviewer in the matching (*M* = 5.73, *SD* = 0.98) compared to the nonmatching condition (*M* = 5.08, *SD* = 1.37; β=.32,t=3.53,p<.001,d=.54, 95%CI[0.26,0.83] ), while for the competitive interaction, there was no difference in feelings of being understood between the matching (*M* = 2.49, *SD* = 1.38) versus the nonmatching condition (*M* = 2.58, *SD* = 1.4115; β=−0.047,t=−0.51,p=.61,d=0.07,[−0.21,0.34]). These results go in line with *H1* (matching of motivational frames would lead to more positive interaction outcomes) and partially in line with *H3* (motivational frame matching would lead to more positive interaction outcomes for a cooperative orientation but lead to less positive interaction outcomes for a competitive orientation).

#### Identification with interviewer

There was a significant main effect of frame, $F\left(1,\;\;391\right)=8.27,\;\;p<.001,\;\;\eta_{2}^{p}=.06,\;$ 95%CI[0.02,1.00] , as well as a main effect of orientation, $F\left(1,\;\;391\right)=124.97,\;\;p<.001,\;\;\eta_{2}^{p}=.24,\;\;[0.18,\;\;1.00]$ . Furthermore, there was a significant interaction effect between frame and orientation on the tendency to identify with the interviewer, $F\left(3,\;\;391\right)=5.65,\;\;p<.001,\;\;\eta_{2}^{p}=.04,$ [0.01,1.00] .

As before, participants were more willing to identify with the interviewer in the cooperative (*M* = 5.08, *SD* = 1.02) compared to the competitive interaction (*M* = 2.60, *SD* = 1.32; β=1.24,t=21.20,p<.001,d=2.11, 95%CI[1.86,2.36] ), lending support for *H2* (a friendly and positive interaction between the investigative interviewer and suspect would lead to more positive interaction outcomes).

Using simple effects tests (see note 6), it was found that, within a cooperative interaction, motivational frame matching did not lead to a higher tendency to identify with the interviewer (*M* = 5.20, *SD* = 1.01), compared to the nonmatching condition (*M* = 4.96, *SD* = 1.02; β=.12,t=1.45,p=.15,d=0.24,95%CI[−0.04,0.52] ), not supporting *H1* (matching of motivational frames would lead to more positive interaction outcomes). However, within a competitive interaction, motivational frame matching did lead to significantly less tendency to identify with the interviewer (*M* = 2.42, *SD* = 1.22) in comparison with the nonmatching condition (*M* = 2.77, *SD* = 1.40; β=−.17, t=−2.09,p=.037,d=0.27,[−0.02,0.55] ). This gives partial support for *H3* (motivational frame matching would lead to more positive interaction outcomes for a cooperative interaction but lead to less positive interaction outcomes for a competitive interaction).

#### Interviewer trust

While there was no significant main effect of frame, $F\left(3,\;\;391\right)=2.41,\;\;p=.07,\;\;\eta_{2}^{p}=.02,$ 95%CI[0.00,1.00] , there was a main effect of orientation, $F\left(1,\;391\right)=42.95,\;\;p<.001,\;\;\eta_{2}^{p}=.10,\;\;[0.06,\;\;1.00]$ , with higher intentions to trust the interviewer in the cooperative (*M* = 4.60, *SD* = 1.26) than the competitive interaction (*M* = 2.52, *SD* = 1.22; β=1.039,t=16.81,p<.001, d=1.67,[1.44,1.90] ). This provides support for *H2* (a friendly and positive interaction between the investigative interviewer and suspect would lead to more positive interaction outcomes). There was also a significant interaction effect between frame and orientation, F(3,391)=3.84, $\;p=.0099,\;\;\eta_{2}^{p}=.03,\;\;[0.00,\;\;1.00]$ .

To explore the interaction further, simple effects tests (see note 6) were used. However, while the overall interaction was significant, the individual tests demonstrated that there was no significant difference between the matching (*M* = 4.75, *SD* = 1.14) and nonmatching conditions (*M* = 4.44, *SD* = 1.37) for both the cooperative (β=.15,t=1.75,p=.081,d=0.24,95%CI[−0.04,0.52] ), and competitive interactions (matching: *M* = 2.34, *SD* = 1.12; nonmatching: *M* = 2.69, *SD* = 1.29; β=−.17, t=−1.94,p=.053,d=.28,[0.00,0.56]) , providing no support for *H1* (matching of motivational frames would lead to more positive interaction outcomes) or *H3* (motivational frame matching would lead to more positive interaction outcomes for a cooperative interaction but lead to less positive interaction outcomes for a competitive interaction).

### Discussion Experiment 2

The aim of Experiment 2 was to validate and replicate the results from Experiment 1 but using a video investigative interview instead of a script-based interview. The results between the two experiments were largely congruent, with matching leading to more positive interaction outcomes within a cooperative interaction (significant dependent variables: willingness to cooperate & feelings of being understood by the interviewer), but less positive interaction outcomes in a competitive interaction (significant dependent variables: willingness to cooperate & identify with the interviewer). Similar to the first experiment, the cooperative interview consistently led to more positive interaction outcomes on all outcome variables compared to when the interview was competitive.

## General discussion

The current paper sought to establish an initial evidence base of the influence of motivational frame matching on participants’ willingness to cooperate and provide information, as well as their perceptions of feeling understood, identifying and trusting an investigative interviewer. Across two experiments, one script-based and another video-based, we found that within a cooperative interaction, motivational frame matching led to a higher willingness among participants to cooperate and to feeling more understood by the interviewer. In contrast, within a competitive interaction, motivational frame matching led to a decrease in the willingness among participants to cooperate and identify with the interviewer. This gives support for the hypothesised interaction between motivational frame matching and the orientation taken towards the interaction. It is consistent with previous language style matching research (e.g. Ireland & Henderson, 2014) and a social engagement theory of matching (Dalton et al., 2010). The positive effects of motivational frame matching also supports previous correlational research from crisis negotiations (e.g. Ormerod et al., 2008).

However, our findings provide a more nuanced picture than previous research. For example, Ormerod et al. (2008) found that motivational frame matching was associated with positive negotiation outcomes, regardless of whether the interaction was cooperative or competitive. We found that matching seems not to be ubiquitously positive, but to interact with the orientation taken towards the interaction. However, an important difference between our study and the study by Ormerod et al. (2008) is that the interactions were all balanced on the orientation dimension. In real interactions (such as in Ormerod et al., 2008) it is arguably rare for dialogue to be consistently competitive or cooperative in nature. Instead, the suspect and interviewer might occasionally take a cooperative orientation in an effort to display basic amiability towards each other. This could help explain some of the differences in the results between the two studies.

While previous research has demonstrated a positive association between language style matching and confessions in interrogations (Richardson et al., 2014), this is the first study so far to have established the positive effects of motivational frame matching in an investigative interviewing context. This is important as motivational frame matching might be somewhat easier to train to investigative interviewers and law enforcement investigators compared to language style matching. For example, elements of motivational frame matching have already been successfully taught to the U.S. Air Force Office of Special Investigations (see Brandon et al., 2019). In contrast, the use of function words (which form the basis for calculating a language style matching score) is believed to occur largely unconsciously (Ireland & Pennebaker, 2010), which suggests that it might be more difficult to train interviewers to match a suspect’s language style than motivational frame.

Looking more closely at the pattern of matching and nonmatching across orientations revealed that, while not reaching statistical significance, all the outcome variables showed the same consistent pattern (i.e. more positive interaction outcomes in the matching condition and less positive outcomes in the nonmatching condition for cooperative interviews; less positive interaction outcomes in the matching condition and more positive outcomes in the nonmatching condition for competitive interviews). Furthermore, these tendencies were supported by the omnibus MANOVA analyses, suggesting that the interaction between frame and orientation was stable across outcome variables. However, it is difficult to know the exact reason why only certain outcome variables reached statistical significance in the individual tests. One potential explanation could be that, despite attempts to make the interview as realistic as possible, it might have been too short to reliably create an impression of successful interpersonal sensemaking. Another reason could be the rather diverse sample which might have contributed to an increase in the within group variance (Fern & Monroe, 1996), attenuating some of the positive effects of matching. A final explanation could be that certain outcome variables (e.g. feelings of being understood and listened to) are more closely aligned with the concept of interpersonal sensemaking as conceptualised in the current study, while other outcome variables (e.g. trust) might have been more of an indirect outcome.

In addition to the positive outcomes of motivational frame matching, we also found that a cooperative interview yielded significantly more cooperation and information gain, feelings of being understood, identification and intention to trust the interviewer among participants. This supports previous research demonstrating the beneficial effects of a friendly and positive interaction on information yield and cooperation within investigative interviews (Brandon et al., 2019; Meissner et al., 2015; Russano et al., 2019).

An important feature of the current experimental design was that the suspect-interviewer interactions were balanced in terms of the orientation taken towards the interaction. Specifically, both the suspect and interviewer took either a cooperative or a competitive orientation towards the interaction. This likely helps explain why matching of motivational frames was not beneficial in the competitive interview. In such an interaction, matching would mean that both the suspect and interviewer were arguing around the same topics (Ireland & Henderson, 2014; Taylor, 2002). Hence, one could surmise that their argument would be more insistent compared to if they had not shared the same goals for the interaction (i.e. motivational frames). Relatedly, competitive matching might have led to a type of conflict spiralling. For example, there is some evidence that conflicts may spiral when people reciprocate a competitive orientation (Alison & Alison, 2020), but the current study is the first to show that this could happen within just five utterances.

While the study provides the first experimental evidence of motivational frame matching and its associated positive outcomes, it is not without its limitations. First, having participants adopt the suspect’s position may have reduced the realism of the study and removed the participants from being more actively engaged in the interaction. Although this may have distorted the effects of the manipulation, we argue it would have attenuated rather than magnified the differences between the matching and nonmatching conditions. However, clearly it would be valuable for future research to actively involve the participants in an interaction, to investigate how this might influence the outcomes of motivational frame matching.

Second, it could be theorised that the simulated interview may have been too short to reliably create a sense of motivation or goal in the suspect and interviewer. This could also have made the effects of matching based on these motivations or goals somewhat weaker and may explain why we did not find significant effects for all the outcome variables (although all were in the predicted direction). Additionally, since the current studies used balanced interaction rounds (both suspect and interviewer being fully cooperative or competitive), it will be important to investigate the effects of motivational frame matching for situations in which the suspect and interviewer have different orientations toward the interaction (e.g. competitive suspect-cooperative interviewer). For such interactions, it could be hypothesised that motivational frame matching would lead to greater cooperation and trust, particularly for getting a competitive suspect to start cooperating. These are all interesting avenues for future research.

## Conclusions

In two experiments, involving both script-based and video-based investigative interviews, we found that matching of motivational frames, as conceptualised in the cylinder model (Taylor, 2002), leads a suspect to be more willing to cooperate and provide information, feel understood, identify and trust an investigative interviewer. However, this was moderated by orientation, such that motivational frame matching only led to more positive interaction outcomes in a cooperative orientation interaction, but less positive interaction outcomes in a competitive orientation interaction. These findings suggest that motivational frame matching is linked with some positive interaction outcomes, but that the orientation taken towards the interaction by the suspect and interviewer moderates these relationships. The current study provides the first experimental evidence of the influence of motivational frame matching on investigative interview outcomes.

## Notes

1. This was calculated based on the conversion formula (Cohen, 1988; Lin, 2024, March 10): $f=\frac{d}{2}$ .

2. This was calculated based on the formula $\eta_{p}^{2}=f^{2}/(1+f^{2})$ from Cohen (1988).

3. Since effect size estimates may differ between types of analysis, the above calculations were made to facilitate consistency between effect sizes (Correll et al., 2020).

4. Originally, the ‘willingness to cooperate’ variable mentioned in the pre-registration contained two items (one relating to willingness to cooperate and the other relating to willingness to provide information). However, since cooperation can refer to more things than merely providing information, these two items were separated into two measures in the analysis. This constituted a deviation from the pre-registration.

5. The unequal group sizes were due to participants being randomly assigned to either an instrumental, relational, identity or nonmatching interview.

6. As these tests were pre-registered and each related to an individual hypothesis (i.e. individual testing), they were not subjected to alpha-adjustments as advised by Rubin (2021).

7. Due to violations of the assumption of homoscedasticity, the Box-Cox transformation was performed before running the analysis.
